# Physiotherapy plus conventional treatment versus conventional treatment only in the treatment of functional constipation in children: design of a randomized controlled trial and cost-effectiveness study in primary care

**DOI:** 10.1186/s12887-018-1231-7

**Published:** 2018-07-31

**Authors:** Jojanneke J. G. T. van Summeren, Gea A. Holtman, Yvonne Lisman- van Leeuwen, Lisa E. A. M. Louer, Alice H. C. van Ulsen-Rust, Karin M. Vermeulen, Boudewijn J. Kollen, Janny H. Dekker, Marjolein Y. Berger

**Affiliations:** 1 Department of General Practice and Elderly Care Medicine, University of Groningen, University Medical Center Groningen, Groningen, The Netherlands; 2 Pelvic Physiotherapy, Practices for Pelvic Physiotherapy, Groningen, The Netherlands; 3 Department of Epidemiology, University of Groningen, University Medical Center Groningen, Groningen, the Netherlands

**Keywords:** Constipation, General practitioner, Pelvic floor, Child and adolescent, Family medicine

## Abstract

**Background:**

Our aim was to design a study to evaluate the effectiveness and cost-effectiveness of adding physiotherapy to conventional treatment for children with functional constipation in primary care. Physiotherapy is focusing on improving the coordination between the pelvic floor and abdominal musculature during bowel movement, while conventional treatment is mainly focusing on symptomatic relief of symptoms, therefore, we expect the effects of physiotherapy will be more sustainable than the effects of conventional treatment. In this paper we describe the final study design and how the design was adapted, to overcome recruitment problems.

**Methods:**

We designed a randomized controlled trial of children aged 4–17 years with functional constipation diagnosed by a general practitioner or pediatrician. Children in the intervention group received physiotherapy plus conventional treatment, and those in the control group received conventional treatment only. Follow-up measurements took place at 4 and 8 months. The primary outcome was treatment success defined according to the Rome-III criteria as the absence of functional constipation, with no laxative use. Secondary outcomes were absence of functional constipation irrespective of laxative use, quality of life, global perceived effect, and costs.

Children were recruited from September 2014 to February 2017. Initially, we aimed to include children with recent symptom onset. However, in the first phase of enrollment we were confronted with an unforeseen recruitment problem: many children and their parents refused randomization because physiotherapy was considered too burdensome for the stage of disease. Therefore, we decided to also include children with a longer duration of symptoms. In total 134 children were included.

**Discussion:**

The target number of participants is achieved. Therefore, the results may change thinking about the management of functional constipation in children.

**Trail registration:**

Netherlands Trial Register (NTR4797), registered 8 September 2014.

## Background

Functional constipation (FC) is a common problem in children [1]. Its etiology is multifactorial, involving age, behavior, pelvic floor function, and gastrointestinal motility. Conventional treatment includes education, dietary advice, toilet training, and laxatives [2, 3]. However, despite this multifaceted approach, 50% of children still experience FC after 6–12 months’ treatment with laxatives and 25% have symptoms that persist into adulthood [4, 5]. Therewith FC has not only a major impact on the quality of life of both children and their families but also increases healthcare costs significantly [6–8].

The pelvic floor and abdominal muscles contract and relax in a coordinated manner during bowel movements, and dysfunction of this interaction could be important in the onset and maintenance of FC [9]. Children with FC, either consciously or unconsciously, appear to strain their pelvic floor muscles and fail to relax the external anal sphincter during bowel movements [10, 11]. In addition, reduced trunk stability may preclude achievement of the posture and intra-abdominal pressure required for defecation [10, 12]. Physiotherapy for FC focuses on improving this coordination between the abdominal and pelvic floor musculature [9]. To date, two small clinical trials in specialist care have shown promising results for the effects of physiotherapy in children with FC [13, 14]. Physiotherapy is expected to give optimal results in children with recent symptom onset [4]. Therefore, it could be particularly effective in children presenting to primary care.

We aimed to design a randomized controlled trial to evaluate the effectiveness and cost-effectiveness of physiotherapy plus conventional treatment in comparison to conventional treatment alone for children aged 4–17 years presenting with FC in primary care. We encountered problems in the recruitment of participants that led us to change the original criteria for participant selection. In this paper we therefore start with presenting our final study design. Thereafter, we describe the process of recruiting participants, including the changes implemented in the original study design. Lastly, we evaluate the representativeness of our study population by comparing characteristics of children that participated and refused to participate in this trial.

## Methods

### Design

We designed a randomized controlled trial that had a follow-up period of 8 months, and wherein children were included between September 2014 and March 2017. The trial was approved by the Medical Ethical Board of the University Medical Center of Groningen (METC2013/331) and was registered in the Netherlands Trial Register (NTR4797). Before enrollment we obtained written informed consent from all parent(s). In addition, children aged ≥12 years provided written informed consent themselves.

### Participants

#### Eligibility criteria

**Fig. 1:**
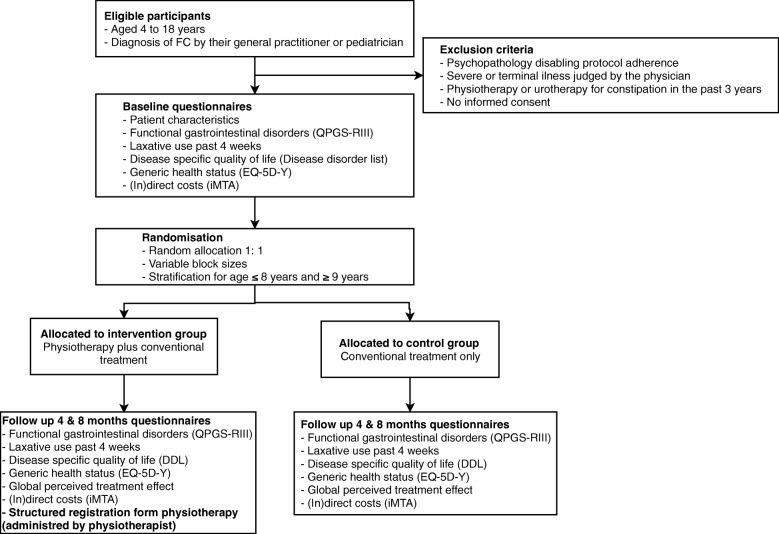
Flowchart of study design: eligibility criteria for participants, planned measurement and timing of baseline characteristics and the primary and secondary outcome measurements. Abbreviations: FC, functional constipation, QPGS-RIII, Questionnaire on Pediatric Gastrointestinal Symptoms Rome-III, EQ-5D-Y, EuroQol-5-dimensions-youth, iMTA, Institute of Medical Technology Assessment Medical Consumption Questionnaire, DDL, Defecation Disorder List

Children were eligible for inclusion if aged 4–17 years and diagnosed with FC by a general practitioner (GP) or general pediatrician. Specifically, children were required to have experienced FC symptoms or to have used laxatives in the 4 weeks before enrollment. Children under the age of 4 years were considered too young to undergo physiotherapy [9]. The exclusion criteria were psychopathology affecting protocol adherence, severe disease (physician determined), and physiotherapy or urotherapy for constipation in the past 3 years (Fig. 1).

#### Patient recruitment

We recruited all children (aged 4–17 years) presenting to general practices or who were newly referred to pediatric outpatient departments with a diagnosis of FC. During the first consultation for FC symptoms, parents and children were informed about this study by their treating physician (incident cases). In addition, any children with a known diagnosis of FC and who had consulted their GP in the past 12 months for this diagnosis were sent a leaflet explaining the study (prevalent cases). Children or their parents (depending on the child’s age) were asked to complete a short questionnaire supplied with the leaflet, detailing whether the child had experienced symptoms of FC or used laxatives in the past 4 weeks. Once completed, they were asked to return the questionnaire.

### Interventions

#### Control group: conventional treatment only

Children in the control group received conventional treatment. No restrictions or recommendations were given to the physicians regarding treatment. However, education, dietary advice, toilet training, and when indicated, laxative prescribing were permitted based on appropriate guidance [2, 3].

#### Intervention group: physiotherapy plus conventional treatment

Children in the intervention group received physiotherapy in addition to conventional treatment. Physiotherapy consisted of a maximum of nine half-hour sessions carried out by specialist physiotherapists, and ended if the physiotherapist considered that treatment was successful or that no more improvement was expected. The physiotherapists were trained to master’s degree level in pediatric or pelvic physiotherapy, and had received postgraduate education in the treatment of bladder and bowel dysfunction in children. The patient-tailored structured treatment program used in this study was developed in cooperation with experienced specialist physiotherapists and approved by all participating specialist physiotherapists before the study. The physiotherapist tailored the treatment program to the individual patient. For each patient and session, the physiotherapists recorded on a structured form the treatment options used to reach the six goals.

In young children, physiotherapy focused on the child and his or her parent(s), while in older children, the focus was mainly on the child. All exercises, materials, and methods were presented in a manner appropriate to the child’s developmental age and locomotor skills. For the patient-tailored structured treatment program used in this study, the six goals were: 1) improving the knowledge about defecation, and the role that the child and/or parent might have in the persistence of symptoms; 2) improving toileting behavior and practicing a stable toilet posture; 3) increasing awareness of the sensation of needing to defecate; 4) learning to relax while defecating; 5) learning to breath correctly to generate adequate intra-abdominal pressure for defecation; and 6) teaching effective straining for defecation. Biofeedback and electrotherapy were not allowed in this study because there is insufficient evidence of their efficacy in children with FC and because we considered these therapies too invasive for treatment of children presenting to primary care [15].

### Randomization and blinding

Children were randomly allocated in a 1:1 ratio to the two treatment groups, using a computer-generated randomization list with random block sizes. Randomization was stratified into those aged 4–8 years and those aged 9–17 years. The randomization list was maintained by a researcher who was not involved in the study and had no access to the allocation site.

Children, parents, physicians, and physiotherapists could not be blinded to the intervention. The investigator was blinded to the assigned study group during data entry and statistical analyses.

### Outcomes

The primary outcome was treatment success, defined according to the Rome-III criteria as the absence of FC without laxative use (see Table 1 for the Rome-III criteria used to define FC) [16]. Thus, a successfully treated child was required to fulfill none or one of the six Rome-III criteria. Other secondary outcomes were absence of FC according to the Rome-III criteria irrespective of laxative use, quality of life, global perceived treatment effect, and costs.

**Table 1:** Description of the Rome III criteria for functional constipation [16]

According to the ROME III criteria, a child must have a developmental age of at least 4 years and fulfill two or more of the following criteria, at least two months prior to diagnosis^a^:	
1) two or fewer defecations in the toilet per week,	
2) at least one episode of fecal incontinence per week,	
3) history of retentive posturing or excessive volitional stool retention at least once a week,	
4) history of painful or hard bowel movement at least once a week,	
5) presence of a large fecal mass in the rectum at least once a week,	
6) history of large diameter stools that may obstruct the toilet at least once a week.	

^a^For the purpose of this study, patients were eligible for enrollment if symptoms were present for at least one month before diagnosis, rather than two months, which is in agreement with the recently published Rome-IV criteria [27]

### Measurements

Figure 1 gives an overview of the measurement and timing of baseline characteristics and the primary and secondary outcome parameters; follow-up measurements took place after 4 and 8 months. We collected the following data at baseline: age, gender, symptom duration, age at symptom onset, symptom chronicity, and whether lower urinary tract symptoms were present. Symptom chronicity was defined as continuous or regular laxative use (≥3 periods) in the 12 months before inclusion.

#### Measurement of the primary outcome

The presence of FC was assessed with a Dutch version of the Questionnaire on Pediatric Gastrointestinal Symptoms Rome-III (QPGS-RIII) [16]. This standardized questionnaire was used to assess if children have experienced functional gastrointestinal symptoms over the last 2 months. We adapted the questionnaire and evaluated symptoms over a period of 4 weeks. Children completed the questionnaire themselves if they were aged 13–17 years, but parents completed the questionnaire if their child was aged 4–12 years. In addition, parents answered the question “Did your child use laxatives in the past four weeks?” (yes or no). If one or more responses were missing for the primary outcome measure, we contacted the child or parent by telephone to obtain an answer.

#### Measurement of secondary outcomes

Disease-specific quality of life was measured with the Defecation Disorder List (DDL), [17, 18] adapted to include only the emotional and social functioning subdomains. The constipation-related and treatment/intervention subdomains were omitted because it has previously been demonstrated that these have low internal consistency and potentially low validity when used to measure disease-specific quality of life [17, 18]. Health status was measured with the EuroQol-5-dimensions-youth (EQ-5D-Y) [19]. Proxy report versions of the DDL and EQ5D-Y questionnaires were completed by parents, and children aged ≥8 years also completed child self-reports. The global perceived treatment effect of patients (GPE) was scored by parents and measured with a 9-point Likert-type scale (very much, much, reasonable, and slightly improved; no change; slightly, reasonable, much, and very much worse). When parents reported that the symptoms of their child were improved very much or much we defined the treatment as successful.

Healthcare consumption related to FC, such as GP or pediatrician visits, drug treatment, and parental productivity loss, were measured with versions of the Institute of Medical Technology Assessment Medical Consumption Questionnaire (iMTA-MCQ) and the Productivity Costs Questionnaire (iMTA-PCQ), respectively, adjusted for FC [20, 21]. Both cost questionnaires were completed by parents only.

If questionnaires were not returned, participants were sent a reminder e-mail after 2 weeks and received a reminder telephone call after 3 weeks.

### Sample size

Sample size estimates were based on a systematic literature review showing that after 6 to 12 months of conventional treatment, 50% of the children were free of symptoms without using laxatives [22]. Physiotherapy in one study has been shown to improve outcomes by 30% compared with conventional treatment alone in children with FC referred to pediatric specialist care [14]. However, that study may have overestimated the magnitude of effect because it was small and underpowered [23]. Therefore, we used a more conservative estimate of the difference in treatment success (25%) between the intervention and control group. The sample size was calculated with expected treatment success rates after 6–12 months of 50 and 75% in the conventional and intervention groups, respectively [14, 22]. Given an expected loss to follow-up of 10%, we estimated a total sample size of 128 children (alpha 0.05, power 0.80).

### Statistical analyses

We will use appropriate descriptive statistics to describe patient characteristics, baseline questionnaire scores, and the proportions of successfully treated children at 4 and 8 months in the intervention and control groups.

We will use multilevel analyses to investigate the longitudinal relationship between the intervention group (physiotherapy plus conventional treatment) and the control group (conventional treatment) with respect to the primary and secondary outcome variables. The applied levels will be repeated measures (that is, time), and patient. We will base our analyses on intention to treat (ITT). The ITT population will consist of all patients who have given informed consent and have been randomly allocated to one of the two treatments, irrespective of whether they received the allocated treatment or not. An additional secondary per protocol analysis (PP) will be conducted for the outcome variable. The PP population will consist of all children randomized in the intervention group receiving at least one physiotherapy session and all children in the control group that had no physiotherapy. Finally, in a sensitivity analysis we will evaluate whether the effect of the intervention is different for children with and without chronic symptoms.

### Economic evaluation

A cost-effectiveness analysis is planned. The primary aim will be to estimate the societal costs, and the secondary aim will be to estimate the cost-effectiveness of treatment with physiotherapy plus conventional treatment compared to conventional treatment alone. In addition, we will perform a cost-utility analysis based on EuroQol-defined utilities. The parental version of the EQ5D-Y questionnaire will be used for these evaluations. The cost-effectiveness analyses will then be expressed as incremental cost-effectiveness ratios (ICERs), displaying the extra treatment costs of physiotherapy to gain one extra patient with successful treatment, as compared with conventional care. In turn, cost-utility analyses will be expressed as incremental cost-utility ratios (ICURs), displaying the extra costs to gain one additional quality-adjusted life year (QALY). Given that the study follow-up was only planned to be 8 months, we will not include discounting of costs and effects. Bootstrap resampling will be performed on the cost and effect pairs to estimate confidence intervals more accurately and to create a cost-effectiveness plane.

## Process evaluation of adaptations to the original study design

### Criteria for participant eligibility

We had intended to include only those children with FC of recent onset. Therefore, we originally excluded children who were using laxatives or who had used laxatives in the previous 3 months. However, when study enrollment started in September 2014, we were confronted unexpectedly with the fact that many children and parents refused to participate in this trial because they considered the symptoms were not severe enough to justify referral for physiotherapy, which could occur if they consented in randomization. Consequently, many of these patients preferred to opt for laxatives before considering referral to physiotherapy. After recruiting only 20 children over a 12-month period, we decided to expand our eligibility criteria to include also those children who were currently using, or who had used, laxatives in the previous 3 months. This meant that our study population was expanded with children with more advanced FC. Expanding the inclusion criteria also allowed us to include children who had been seen by their GP for FC in the past 12 months, as well as consecutive children newly referred to pediatric outpatient departments. For budgetary reasons, the delay in participant recruitment forced us to shorten the planned follow-up period from 12 months to 8 months. The Medical Ethical Board of the University Medical Center of Groningen approved these changes in study design (METC2013/331).

### Sample size calculation

The original sample size calculation was based on conventional treatment being successful in 60% of children consulting their GP for the first time for FC [24]. At that time, no studies had reported on the treatment effects of physiotherapy, and we estimated a 20% difference in treatment success between the intervention and control groups to be relevant [22]. Thus, we expected the treatment under study would be successful in 80% of the children receiving physiotherapy. Given an expected loss to follow-up of 10%, we had calculated that 180 children would be required for the study (alpha 0.05, power 0.80). However, since the original design, a study had been reported on the effectiveness of physiotherapy in childhood FC in a pediatric outpatient department [14]. Therefore, coupled with the changes in study design, we reconsidered our sample size calculation (see methods section).

### Representativeness of the finally selected study population

**Fig. 2:**
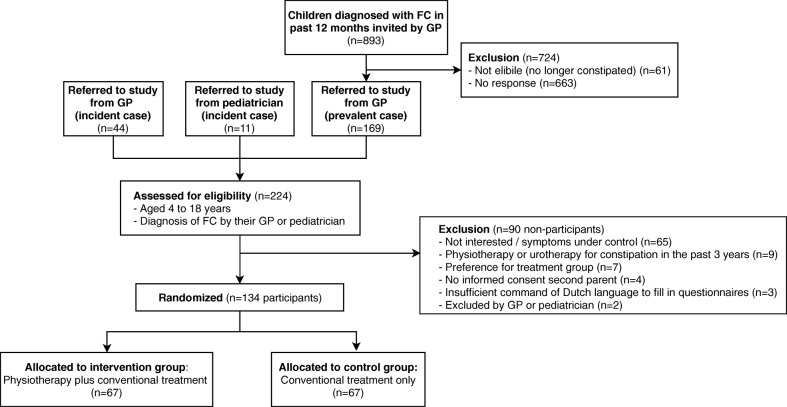
Flowchart of actual participant recruitment and participant flow. Abbreviations: FC, Functional constipation, GP, General practitioner

Children were recruited from 93 general practices (209 GPs) and 5 general pediatric outpatient departments in district hospitals between September 2014 and March 2017. Of the 224 children assessed for eligibility, 213 children were invited by GPs: 44 children with a new diagnosis (incident cases), and 169 children with a diagnosis of FC within the past 12 months (prevalent cases); and 11 newly referred children were invited by pediatricians (Fig. 2).

We compared patient characteristics between children included in the trial (participants, *n* = 134) and children who refused to participate or who met the exclusion criteria (non-participants, *n* = 90) (Fig. 2 and Table 2). Participants were found to be slightly younger (mean age, 7.5 ± 3.5 years) compared with non-participants (mean age, 8.2 ± 3.8 years), but the boy-to-girl ratio was comparable (Table 2). Among non-participants, symptom chronicity was only assessed in children referred as prevalent cases by their GP for logistical reasons. Comparing chronicity among prevalent cases seems to show that participants more often had chronic symptoms at baseline (65%) compared with non-participants (43%) (Table 2).

**Table 2:** Characteristics of participants and non-participants

	Participants (*N* = 134)	Non-participants (*N* = 90)
Age (mean, SD)	7.5 ± 3.46	8.23 ± 3.80^a^
Gender (% girls)	61.2	60.0^a^
Referred to study by:		
- GP (incident case), (n, %)	22 (17%)	22 (24%)
- Pediatrician (incident case), (n, %)	6 (4%)	5 (6%)
- GP (prevalent case), (n, %)	106 (79%)	63 (70%)
Chronicity of symptoms before randomization ^b, c^		
-Yes (n, %)	67 (65%)	16 (43%)
-No (n, %)	36 (35%)	21 (57%)

*GP* General practitioner, *SD* standard deviation

^a^ Age and gender were not available of 19 non-participants

^b^ Comparison of chronicity of symptoms between participants and non-participants, was only performed for prevalent cases in whom the question about chronicity was asked (participants *n* = 103, non-participants *n* = 37)

^c^ Symptom chronicity was defined as continuous or regular laxative use (≥3 periods) in the 12 months before inclusion

## Discussion

Although two small clinical trials have shown that physiotherapy for FC could be a promising treatment for children in specialist care, [13, 14] we are not aware of any trial evaluating its effectiveness in primary care where most children with FC are diagnosed and treated [25]. The aim of physiotherapy is to improve the coordination between the abdominal and pelvic floor musculature during bowel movement [9]. The strength of physiotherapy is that physical exercises are combined with cognitive and behavioral elements, such as education and toilet training [26]. Treatment guidelines recommend that these cognitive and behavioral elements be discussed by doctors during a consultation [2, 3]. However, this might be problematic because GPs focus on symptomatic relief of symptoms. In addition, the consultation time is only 10 min in primary care, which limits the time for proper education, and advices on toilet training.

Initially, we had aimed to assess physiotherapy in children with recent-onset FC, for two main reasons. First, we assume that the long-term prognosis could be more improved if children receive treatment early in the disease process [4]. Second, we think that duration of symptoms and of treatments would be more homogenous in children with recent onset of symptoms. However, we discovered that parents and children could not be motivated for a time-intensive therapy like physiotherapy for symptoms they considered to be temporary and mild. Our subsequent comparison of participants and non-participants confirmed that children were more inclined to participate if they had longer symptom durations and regular laxative use. Therefore, our study will generate results on the effects of physiotherapy for children with more advanced FC than we had originally planned. Specifically, we expect our results to concern those cases where the child or parent have experienced conventional primary care treatment to be insufficient.

We hypothesized that physiotherapy, by increasing awareness of the abdominal and pelvic floor muscle use during defecation, would have a more sustained effect on outcomes than symptomatic treatment with laxatives. Although we were therefore particularly interested in the long-term effects of physiotherapy, the follow-up duration had to be shortened from 12 to 8 months. However, a follow-up duration of 12 months is probably also too short to evaluate whether the effects of physiotherapy are sustainable. The results of this study will help deciding if it is justified to plan a long term follow-up study.

### Clinical impact

We designed the first trial to evaluate the effectiveness of physiotherapy as a first-line treatment for childhood FC in primary care. In total 134 children were included, giving this study sufficient power to lead to promising results. These results may change thinking about the management of functional constipation in children.

## Abbreviations

DDL: Defecation disorder list
EQ5D-Y: EuroQol-5-dimensions-youth
FC: Functional constipation
GP: General practitioner
GPE: Global perceived effect
ICER: Incremental cost-effectiveness ratio
ICUR: Incremental cost-utility ratio
iMTA-MCQ: Institute of Medical Technology Assessment Medical Consumption Questionnaire
iMTA-PCQ: Institute of Medical Technology Assessment Productivity Costs Questionnaire
ITT: Intention to treat
PP: Per protocol
QALY: Quality-adjusted life year
QPGS-RIII: Questionnaire on Pediatric Gastrointestinal Symptoms Rome-III
